# ToxReporter: viewing the genome through the eyes of a toxicologist

**DOI:** 10.1093/database/baw141

**Published:** 2016-10-02

**Authors:** Mark Gosink

**Affiliations:** Department of Investigative Toxicology, Pfizer Inc, Eastern Point Rd, MS 8274-1246, Groton, CT 06340, USA

## Abstract

One of the many roles of a toxicologist is to determine if an observed adverse event (AE) is related to a previously unrecognized function of a given gene/protein. Towards that end, he or she will search a variety of public and propriety databases for information linking that protein to the observed AE. However, these databases tend to present all available information about a protein, which can be overwhelming, limiting the ability to find information about the specific toxicity being investigated. ToxReporter compiles information from a broad selection of resources and limits display of the information to user-selected areas of interest. ToxReporter is a PERL-based web-application which utilizes a MySQL database to streamline this process by categorizing public and proprietary domain-derived information into predefined safety categories according to a customizable lexicon. Users can view gene information that is ‘red-flagged’ according to the safety issue under investigation. ToxReporter also uses a scoring system based on relative counts of the red-flags to rank all genes for the amount of information pertaining to each safety issue and to display their scored ranking as an easily interpretable ‘Tox-At-A-Glance’ chart. Although ToxReporter was originally developed to display safety information, its flexible design could easily be adapted to display disease information as well.

**Database URL:** ToxReporter is freely available at https://github.com/mgosink/ToxReporter.

## Introduction

It is becoming standard practice within the pharmaceutical industry to evaluate new gene targets for their potential safety risks very early in the drug discovery process before compound development begins (1). Such an evaluation can save both time and money by allowing priorities to be made among otherwise equally valid targets and by identifying potential assays and activities that can be performed concurrently with early efficacy studies. By performing this work early on, the risk of significant costs in time and money are lessened should the target eventually fail due to safety issues. Such activities are often referred to as de-risking efforts. However up-front target safety evaluations require time-consuming searches of wide-ranging databases from literature reports to genetic mutations to gene annotations. Each of these sources needs to be evaluated in terms of the types of risks in order that the appropriate de-risking activities can be scheduled.

ToxReporter is a web-based application that streamlines this process by categorizing public and proprietary domain-derived information into predefined safety categories according to a customizable lexicon. This approach allows the user to quickly focus (via ‘red-flags’) on annotation information within specific areas of safety concern which are associated with a gene. For example, when examining cardiovascular (CV) issues, ToxReporter flags links to related PubMed articles spanning cardiac topics ranging from ‘Aneurysm’ to ‘Ventricular Remodeling’ while links to articles related to other safety categories remain hidden.

ToxReporter also uses a scoring system based on these red-flags to rank the relative amount of safety issue related information for individual genes versus all other genes in the genome and to display these Safety Lexicon (SL) scores as an easily interpretable ‘Tox-At-A-Glance’ chart. The SL scores within ToxReporter are not meant to be predictive of safety risk but rather help identify areas of potential concern based on the associated data. The safety categories used within ToxReporter are based on the SL, a controlled vocabulary of a number of organ and mechanism-related areas of drug attrition developed at Pfizer to help understand toxicity mechanisms (see online Supplementary Materials). Both Pfizer’s SL and mappings of SL terms to many public classifications are included with the loading scripts. A complete set of the loading instructions including links to the third party external data sources is provided in the documentation folder from the ToxReporter github site. Other ontologies or even multiple ontologies could be implemented; however they would have to be mapped to the public classification systems using an included classification mapping script.

## Materials and Methods

ToxReporter is written in PERL and JavaScript and is hosted on a Linux server running Apache 2.2.15 (Red Hat) Server. Data in ToxReporter is housed in a MySQL (version 5.5.20) database (http://www.mysql.com/). The data loading phase of ToxReport requires the running of a number of PERL scripts to populate the database and create associations between gene annotations and the SL terms. Usage of ToxReporter tool occurs via cgi scripts running on the web-server. A complete set of the PERL scripts, the SL ontology, and the ontology mapping information needed to create the database and to load the data can be downloaded from: https://github.com/mgosink/ToxReporter. An enhanced entity–relationship diagram of the ToxReporter database is available as a supplementary Materials (see online Supplementary Figure S1).

### Data loading

Gene annotation information is downloaded from a wide variety of resources and loaded into the ToxReporter database. These gene annotation resources include Gene ID, Taxonomy ID, Homologene ID, Online Mendelian Inheritance in Man (OMIM) IDs, Gene Ontology IDs, and gene name aliases from NCBI’s Gene database (2). Potential dominant mutations in OMIM are extracted by manually querying the OMIM interface for dominants, incomplete penetrance or variable expression in the clinical synopsis field. The resulting list of OMIM IDs is then loaded into ToxReporter as potential dominants (3). The Gene Ontology structure is downloaded from the Gene Ontology Consortium (4). ToxReporter also extracts the Medical Subject Headings (MeSH) for all articles in the Medline subset of PubMed (2). Non-synonymous SNP frequency information is downloaded from the Exome Variant Server (5). Phenotype data for mouse gene mutation as well as the Mammalian Phenotype Vocabulary is extracted from the Mouse Genome Informatics at the Jackson Laboratory (6). Gene to pathway data is extracted from Ingenuity Pathways (IPA QIAGEN Redwood City, www.qiagen.com/ingenuity). Tissue specificity expression information is extracted from gene expression [GDS592 and GDS596] in the Gene Expression Omnibus (7). ToxReporter also extracts human genetic information and corresponding PubMed articles from the Genetic Association (GA) database (8), and the Genome-Wide Association Study database (GWAS) (9).

**Figure 1.**
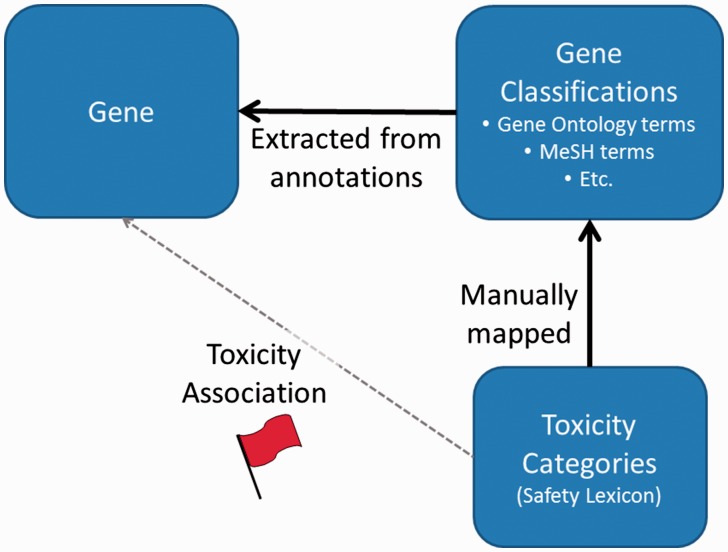
General process for association of ‘red-flags’ with genes. Gene annotation terms are extracted from multiple sources. Independently, ToxReporter contains mappings between many gene classification terms and toxicity categories (i.e. SL) which are used to infer associations between the toxicity classifications and the genes. If a gene is searched in context of a toxicology term then the corresponding gene annotations terms are high-lighted with a red-flag. Other gene annotation terms are hidden from view.

Once third party data has been installed, toxicity categories are then linked to individual genes based on mappings between gene annotation categories and the toxicity categories (Figure 1). The ToxReporter database is structured so that other data sources can be added with only minimal programming effort. ToxReporter’s database is centered on the gene entities with most of the other above extracted information organized as hierarchical classifications of the genes (see database diagram in online Supplementary Materials). At Pfizer, these classifications were manually annotated for their relationships to an internally generated safety-related vocabulary (the SL). By mapping the classifications to SL terms, genes with these classifications can receive a ‘red-flag’ high-lighting the connection between a gene and a safety-related classification term.

In the case of tissue expression profiles, direct mappings between SL term and tissue types were individually created. GO terms, mouse phenotypes and MeSH terms are organized into hierarchical ontologies. For these categories, mappings between SL terms and a number of higher level ontology terms were created manually and any ontology child terms were inferred to have the parent mapping. For MeSH terms, there is the added caveat that a gene must be linked to three or more articles which are annotated with the MeSH term to avoid spurious linkages. The number of articles required to link a gene with a MeSH term were chosen empirically however it can be adjusted during the loading process.

OMIM, GA, and GWAS annotations are linked to SL terms based on their referencing publications. These annotations are red-flagged if they are linked to PubMed articles which are, in turn, linked to MeSH term(s) which had been red-flagged above. For example, an OMIM entry would be categorized as being linked to the above ‘Cardiovascular_System’ term if it cited PubMed article(s) which had been annotated with the MeSH term ‘Heart Diseases’.

Pathways are linked to SL terms via a gene set enrichment (10) type approach using the above red-flagged mouse phenotype data. Genes from each pathway were analyzed by Fisher’s exact test against gene-sets created from each mouse phenotype. If the genes in a pathway showed a *q*-value corrected significance less than 1e−5 and had an overlap of 5 or more genes with a red-flagged phenotype, the pathway was red-flagged with the same SL term(s).

In the final loading step, the number of red-flags or inferred associations between individual genes and the various categorized data sources are counted and scored to establish the relative association between the individual genes and the SL term. Scoring is not a measure of relative risk but rather reflects the relative amount of information associated between a given gene and a safety term as compared with other genes in the genome. SL scores are the percentile ranking of the log10 of the number of red-flagged terms for a given gene versus all other genes in that species genome. Log10 values are used to normalize the data because of the number of red-flags per gene follows an exponential distribution. As implemented, the number of red-flags is weighted based on the needs of toxicologists at Pfizer. (For example, at Pfizer, human genetic information is emphasized so is weighted 2× other flags while tissue expression is less emphasized so expression red-flags are weighted at 1/5× other flags.) However, the weighting can be easily altered within the loading script. The individual SL scores are calculated from the weighted counts as follows:

Safety Lexicon Score=(GFC−MinFC) / (MaxFC−MinFC)

Where GFC = log10 of the total number of red-flags for current gene in current safety category; MinFC = log10 of the minimum number of flags for any gene in current safety category (zero count genes are not used); and MaxFC = log10 of the maximum number of flags for any gene in current safety category.

### Tool usage

**Figure 2.**
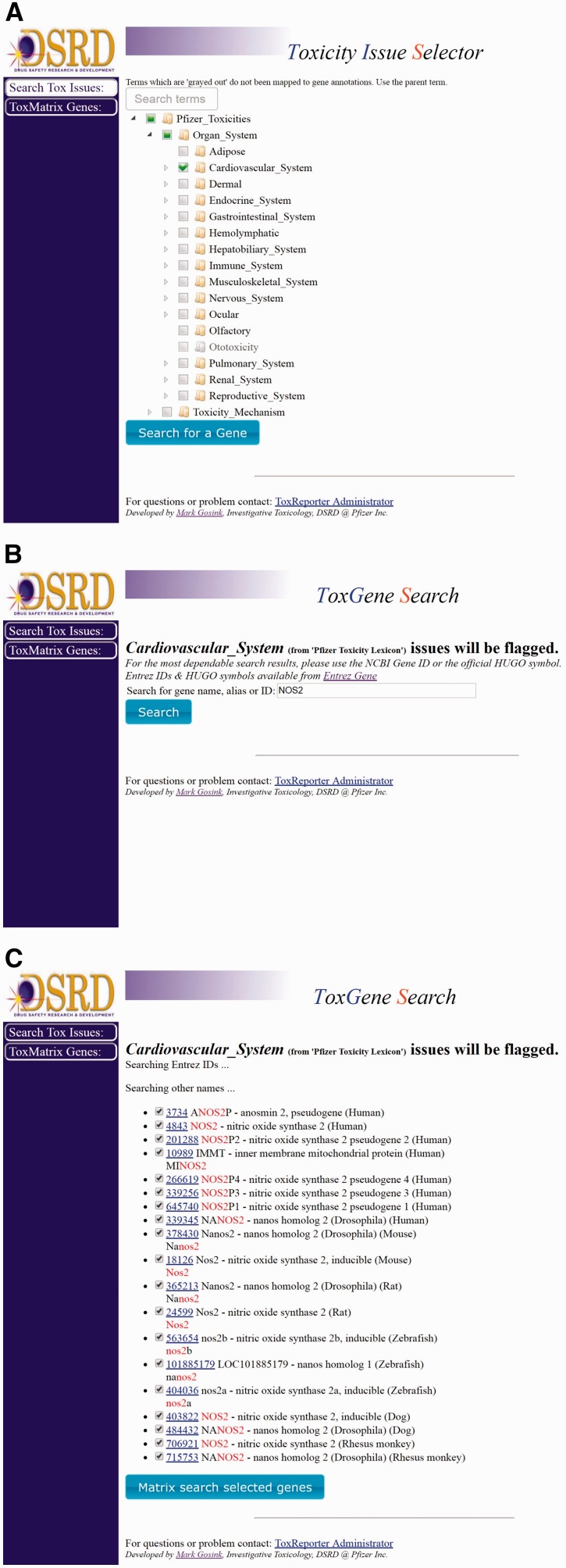
Typical use workflow. **(A)** To search ToxReporter, the user begins by checking the box next to an area of interest from the SL ontology. **(B)** Once a category has been chosen, genes can be searched by name, symbol or NCBI Gene ID. **(C)** Search will return all genes with matching hits high-lighted in red. Clicking on the gene’s link will open the ToxReporter Gene View.

**Figure 3.**
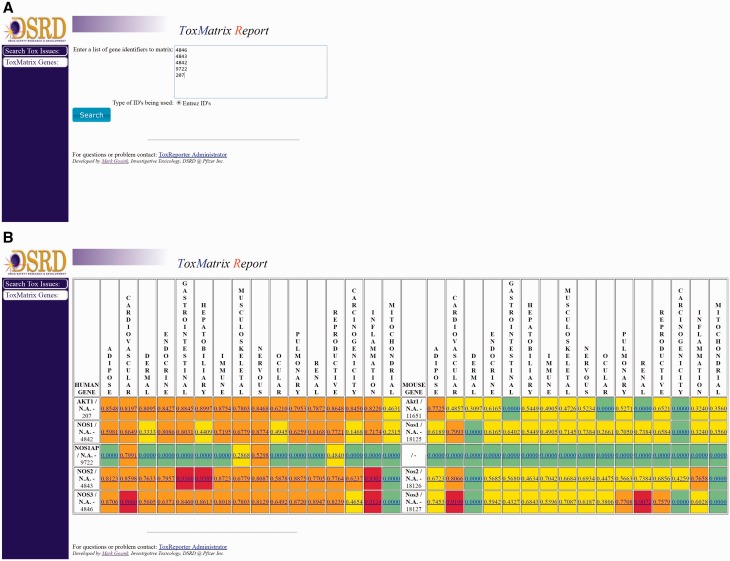
Multiple gene analysis workflow. To search multiple genes simultaneously, the user clicks on the ‘ToxMatrix Genes’ button. **(A)** This action opens a new page where the user can then submit a list of NCBI Gene IDs. **(B)** Submitting the list will return a heat-map display of all matching human and mouse homologs and their corresponding SL scores. SL scores ≥ 0.9 are high-lighted with red, scores ≥ 0.50 with orange, other positive scores with yellow. SL scores of 0.0 are high-lighted with green and indicate categories for which there are no flagged annotations. Clicking on the gene’s link will open the ToxReporter Gene View.

Genes in ToxReporter can be selected via one of two approaches. The first approach was developed to allow help safety domain experts (i.e. developmental and reproductive toxicology) identify potential links between specific genes and their area of expertise. To use this method, a category is first selected from a ‘tree view’ of all ontology terms (Figure 2A). Next, genes can be searched using their NCBI Gene ID, gene symbol or other aliases (Figure 2B). All matching genes are displayed as a list from which the user can either select a single gene (Figure 2C) or by clicking on the ‘Matrix search selected genes’ button can display a heat-map of the human and mouse homologs versus their SL scores (Figure 3B). With the heat-map, users can select a gene/score to open the ToxReport Gene View for that gene. A second approach to gene selection in ToxReporter was developed for users who either did not wish to select a starting SL term or who had a set of genes to investigate. For this approach, a list of NCBI Gene IDs is submitted to the ToxMatrix function (Figure 3A) and a heat-map of all matching human and mouse homologs versus SL scores is displayed (Figure 3B). Users then click on an individual gene/score to open the corresponding ToxReporter Gene View.

**Figure 4:**
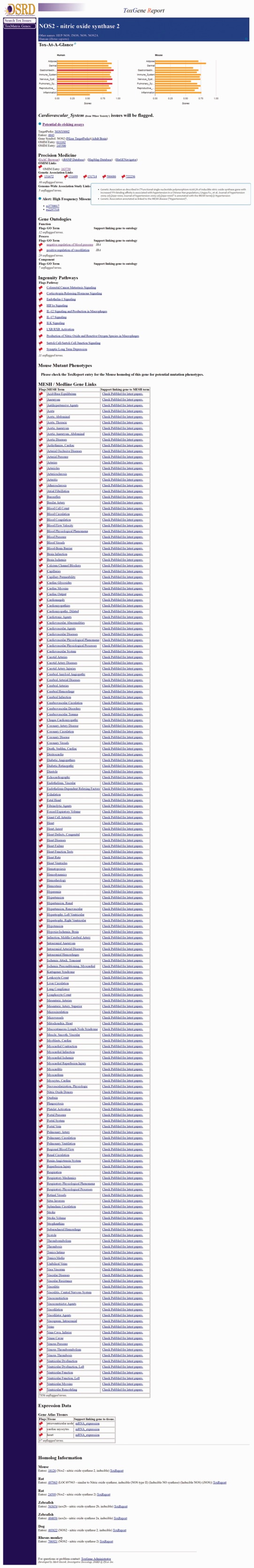
Gene View. An example of the Gene View of CV-system related information for NOS2 is shown. ‘Tox-At-A-Glance’ bar chart at the top of the page can be used to switch category views. Red flags high-light cases where information about the selected gene is associated to the currently selected information category. A mouse-over tooltip for one genetic associated study is shown. Information linked to the gene but not the selected category is noted as ‘unflagged terms’.

**Figure 5:**
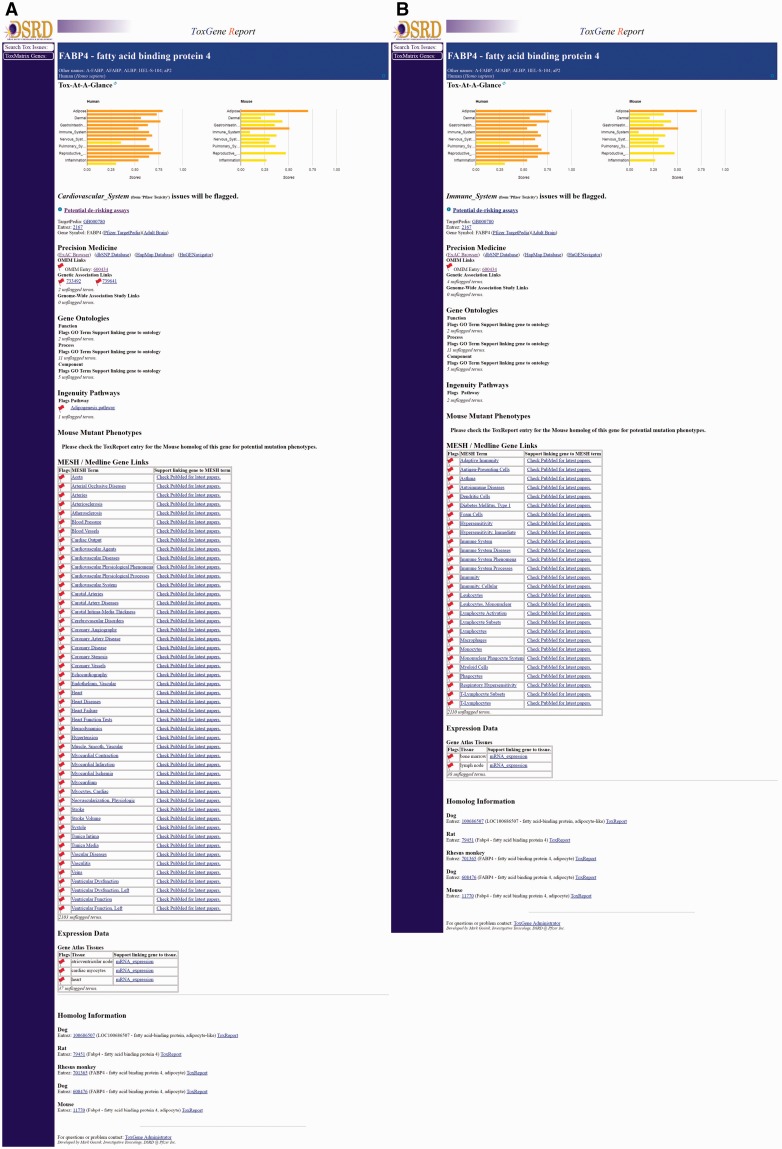
Comparison of gene views. Close-ups of matching parts of the FABP4 Gene View pages for two categories are shown. Panels **(A)** and **(B)** show CV- and IS- related information, respectively. Note: OMIM entry 600434 is flagged in both category views as the OMIM entry references publications describing FABP4’s role in CV disease and other publications describing its role in macrophage function.

When a gene/category combination is selected, the ToxReport Gene View is opened to display information about the gene (Figure 4). At the top of the page is the ‘Tox-At-A-Glance’ bar chart of SL scores that provides an overview of the relative amount of information linking the selected gene and major areas of toxicity concern. For SL scores ≥ 0.9 the graph bar is colored red, SL scores ≥ 0.50 the bar is colored orange and all other SL scores are yellow. Below that are links to basic information about the gene. Below those links are the various information source links that have been mapped to the selected SL term. These sources are highlighted with a ‘red-flag’. Mousing-over the red-flag will display a tooltip popup of information about why that information is red-flagged (Figure 4). The information that is displayed and flagged is specific to the category which was selected by the user. Either selecting a different category at the start or by clicking one of the category bars in the ‘Tox-At-A-Glance’ chart will display different information (Figure 5A and B). Additional information about a gene is noted as unflagged terms but is hidden from view.

## Results and Discussion

### Results

ToxReporter has been in continuous use at Pfizer for over 6 years with dozens of unique users accessing the system each year. It has also been integrated into several other Pfizer applications including the previously published ToxEvaluator (11). The open architecture has permitted the ready addition of new data sources. For example, a precision medicine component was recently added with OMIM, GA and GWAS links based on the associated publications for each.

Currently a number of resources, such as the Comparative Toxicogenomics Database (CTD), DrugBank and ToxEvaluator, are available to the toxicologist to aid in their de-risking efforts to understand the mechanism behind toxic response to a chemical (11–13). However these tools require a compound to have already been identified. ToxReporter was developed to be used before a compound has been identified. ToxReporter’s primary function is to help drug safety and discovery teams to identify potential risks in new target genes. ToxReporter can be used to help understand toxicity mechanisms for compounds; however, in such cases it is used in conjunction with other tools to identify the likely on-target and off-target genes. Once a list of likely targets has been identified, it is submitted to the ToxMatrix function described earlier.

### General use case

As an example of how ToxReporter can save time by permitting focused investigation of a target, we examined what evidence links nitric oxide synthase 2 (NOS2) to the CV system. NOS2 is involved in a large number of cellular processes and searching PubMed yields over 2500 articles about this gene. A toxicologist who was investigating CV issues related to a compound acting on the NOS2 protein would have a difficult time identifying relevant articles. However, ToxReporter identifies the CV-related articles and groups the articles by subject according to their CV-related MeSH terms. A scientist investigating the same gene for immune system (IS)-related effects would see 130 IS-related MeSH terms with linked articles. Similarly, examination of the 196 phenotypes associated with mouse mutations in this gene would show 40 CV and 32 IS phenotype when using CV and IS views, respectively.

### Specific use case

zActivation of the leptin receptor has been shown to induce dramatic weight loss in mice (14). Because of this effect, agonism of the leptin receptor was considered a potential avenue to treat obesity. During the period when an initial evaluation of leptin for weight loss was being evaluated, the literature focused predominantly on leptin’s effects on adipose tissue. However, during the pre-compound development safety review of leptin, ToxReporter identified a potential risk for increased blood pressure (15). Because this potential risk, blood pressure measurement assays were slated to be carried out immediately after efficacy testing. Although leptin treatment has since been shown to be inefficacious for weight loss in humans (16), it has been shown to have a role in the regulation of blood pressure (17).

## Conclusions

ToxReporter was developed to simplify the assessment of new target genes for potential safety issues. By displaying or hiding gene annotation information according to specific areas of drug safety attrition, toxicologists can quickly evaluate the relevancy of those annotations and thereby identify de-risking activities which can be carried out early in drug development. By separating the annotations into specific areas, low frequency annotations which might otherwise been missed are made to stand out. Finally, while ToxReporter is targeted to safety attrition issues, by mapping gene annotations to a disease lexicon, the ToxReporter system could easily be adapted to drug discovery groups.

## Supplementary data

Supplementary data are available at *Database* Online. 

Supplementary Data
