# Supplementary material for: ToxReporter: viewing the genome through the eyes of a toxicologist
Source: Database (Oxford). 2016 Oct 2;2016:baw141. doi: 10.1093/database/baw141 (PMC5199150; doi:10.1093/database/baw141)
Supplement: Supplementary Data [file supp_2016_baw141_index.html]

Supplementary Data 

# ToxReporter: viewing the genome through the eyes of a toxicologist

## Supplementary Data

files

- Supplementary Data - zip file
