# Supplementary figures and images for: ToxReporter: viewing the genome through the eyes of a toxicologist
Source: Database (Oxford). 2016 Oct 2;2016:baw141. doi: 10.1093/database/baw141 (PMC5199150; doi:10.1093/database/baw141)

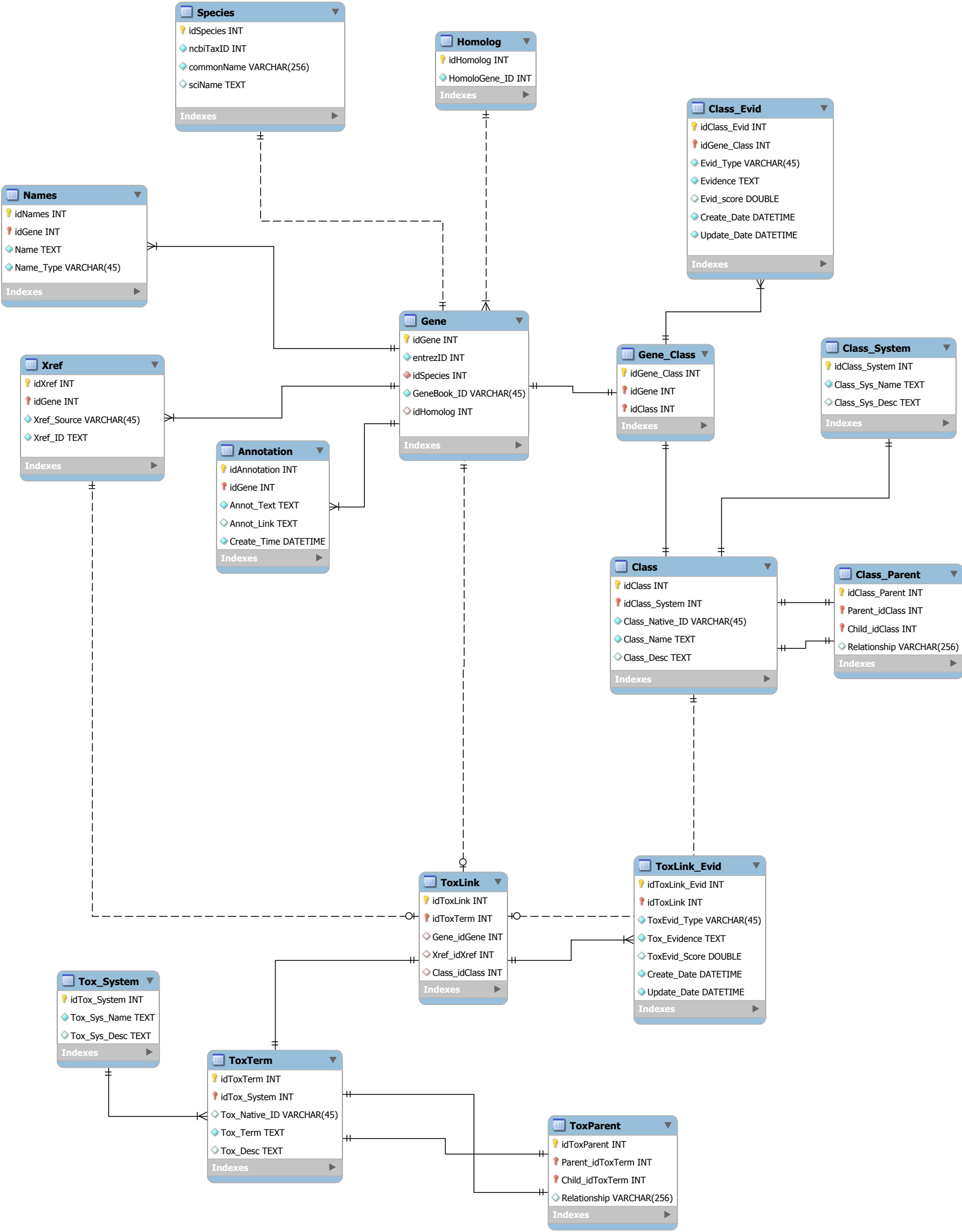

Supplement: Supplementary Data [file supp_baw141_baw141_Supp.zip › Supp Figure S1.pdf]
